# Serotonin Receptor Signaling Promotes Amoeboid Migration of Melanoma Cells Through cAMP/PKA Activation

**DOI:** 10.64898/2026.07.27.741069

**Published:** 2026-07-28

**Authors:** Alleah G. Abrenica, Sylvia Kuang, Neelakshi Kar, Jeremy S. Logue

## Abstract

Melanoma cells frequently utilize bleb-driven amoeboid migration to navigate confined microenvironments during metastasis. Through a morphology-based drug repurposing screen, we identified the anti-migraine medication dihydroergotamine (DHE) as a potent inhibitor of this behavior. We show that DHE suppresses amoeboid motility by targeting serotonin receptors, primarily 5-HTR7. Trace levels of peripheral serotonin activates 5-HTR7, which in turn stimulates cAMP/PKA and ROCK signaling. This signaling cascade increases the cortical contractility necessary to sustain blebbing. Accordingly, DHE treatment or PKA inhibition significantly impairs confined migration without altering cell proliferation. Clinically, elevated levels of serotonin-degrading enzymes, such as MAOB, correlate with favorable patient prognoses and are reduced in metastatic lesions. Ultimately, these findings reveal an unanticipated role for peripheral serotonin in melanoma biomechanics and highlight serotonin receptors as highly actionable targets to limit metastatic dissemination.

**Significance Statement:**

Metastasis is the leading cause of melanoma-related mortality, driven in part by the ability of tumor cells to adopt a highly contractile amoeboid mode of migration that enables movement through spatially confined tissues. This study uncovers a previously unrecognized mechanobiological role for peripheral serotonin in promoting this invasive behavior. We show that low concentrations of serotonin activate cAMP/PKA signaling to generate the actomyosin contractility required for amoeboid migration. Importantly, the FDA-approved migraine drug dihydroergotamine (DHE) blocks this pathway and significantly inhibits cancer cell motility. Supported by patient data linking reduced serotonin degradation with increased metastatic progression, these findings identify serotonin signaling as a novel driver of melanoma dissemination and provide a compelling rationale for repurposing serotonin-targeting therapeutics to prevent metastatic spread.

## Full Text Availability

The license terms selected by the author(s) for this preprint version do not permit archiving in PMC. The full text is available from the preprint server.

